# Safety of colchicine and NSAID prophylaxis when initiating urate-lowering therapy for gout: propensity score-matched cohort studies in the UK Clinical Practice Research Datalink

**DOI:** 10.1136/ard-2023-224154

**Published:** 2023-10-03

**Authors:** Edward Roddy, Ram Bajpai, Harry Forrester, Richard James Partington, Christian D Mallen, Lorna Elise Clarson, Nishita Padmanabhan, Rebecca Whittle, Sara Muller

**Affiliations:** 1 School of Medicine, Keele University, Keele, UK; 2 Haywood Academic Rheumatology Centre, Midlands Partnership University NHS Foundation Trust, Stoke-on-Trent, UK

**Keywords:** Gout, Anti-Inflammatory Agents, Non-Steroidal, Epidemiology

## Abstract

**Objectives:**

To determine the risk of adverse events associated with colchicine or non-steroidal anti-inflammatory drug (NSAID) prophylaxis when initiating allopurinol for gout.

**Methods:**

We conducted two matched retrospective cohort studies in linked UK Clinical Practice Research Datalink and Hospital Episode Statistics datasets. Adults initiating allopurinol for gout with (1) colchicine or (2) NSAID prophylaxis were compared with those initiating without prophylaxis, individually matched by age, sex and propensity to receive the relevant prophylaxis. Weighted Cox proportional hazards models investigated associations between colchicine/NSAID and specified adverse events.

**Results:**

13 945 individuals prescribed colchicine were matched to 13 945 with no prophylaxis and 25 980 prescribed NSAID to 25 980 with no prophylaxis. Adverse event incidence rates were <200/10 000 patient-years except diarrhoea (784.4; 95% CI 694.0 to 886.5) and nausea (208.1; 95% CI 165.4 to 261.7) for colchicine and angina for NSAID (466.6; 95% CI 417.2 to 521.8). Diarrhoea (HR 2.22; 95% CI 1.83 to 2.69), myocardial infarction (MI) (1.55; 95% CI 1.10, 2.17), neuropathy (4.75; 95% CI 1.20 to 18.76), myalgia (2.64; 95% CI 1.45 to 4.81), bone marrow suppression (3.29; 95% CI 1.43 to 7.58) and any adverse event (1.91, 95% CI 1.65 to 2.20) were more common with colchicine than no prophylaxis, but not nausea/vomiting (1.34; 95% CI 0.97 to 1.85). Angina (1.60; 95% CI 1.37 to 1.86), acute kidney injury (1.56; 95% CI 1.20 to 2.03), MI (1.89; 95% CI 1.44 to 2.48), peptic ulcer disease (1.67; 95% CI 1.14 to 2.44) and any adverse event (1.63; 95% CI 1.44 to 1.85) were more common with NSAID than without.

**Conclusions:**

Adverse events were more common when allopurinol was initiated with prophylaxis, particularly diarrhoea with colchicine. Other events were uncommon, providing reassurance for patients and clinicians to enable shared decision-making.

**WHAT IS ALREADY KNOWN ON THIS TOPIC**

- Colchicine or non-steroidal anti-inflammatory drug prophylaxis reduces the incidence of gout flares when initiating urate-lowering therapy. However, the incidence of prophylaxis-related adverse events is not well established.

**WHAT THIS STUDY ADDS**

- Although adverse events were more common when allopurinol was initiated with prophylaxis, adverse events other than diarrhoea were uncommon, providing reassurance for people with gout and clinicians.

**HOW THIS STUDY MIGHT AFFECT RESEARCH, PRACTICE OR POLICY**

- Our findings provide reassurance for people with gout and clinicians about the safety of flare prophylaxis that can inform shared decision-making regarding drug treatment.

## Introduction

Gout is the most common inflammatory arthritis, affecting 2.5% of adults in the UK,^1^ and is associated with significant comorbidity and impairment of health-related quality of life.^2 3^ It is caused by elevation of the serum urate level (hyperuricaemia), which leads to the formation and deposition of monosodium urate (MSU) crystals in and around joints. These crystals then provoke recurrent inflammatory flares of excruciating joint pain and swelling, most commonly affecting the first metatarsophalangeal joint.

The long-term management of gout involves taking urate-lowering therapy (ULT), most commonly allopurinol, to reduce serum urate levels which, over several months, leads to gradual dissolution of MSU crystals and cessation of flares.^4–8^ Although the aim of ULT is to prevent flares, initiation or increasing the dose of ULT often triggers a gout flare, which can lead to ULT being stopped as patients and practitioners may believe that it has worsened the gout.^9^ Gout management guidelines, therefore, recommend coprescription of prophylactic colchicine or non-steroidal anti-inflammatory drugs (NSAIDs) for several months when initiating ULT to prevent ULT-induced flares, and agree that colchicine is the first-line recommended drug for prophylaxis when initiating ULT.^4–7^

Randomised trials demonstrate the effectiveness of colchicine prophylaxis,^10^ which has also been shown to be cost-effective in a cost–utility analysis.^11^ Diarrhoea, nausea and vomiting are common side effects of colchicine, but less is known about the occurrence of more serious side effects such as myopathy, rhabdomyolysis, neuropathy or bone marrow suppression. A meta-analysis of the side effects of colchicine reported in randomised trials identified 35 randomised trials, but only 3 included people with gout receiving colchicine prophylaxis.^12^ While diarrhoea and gastrointestinal events were the most common side effects of colchicine and other more serious side effects were uncommon in the reported trials, it was acknowledged that most included trials were limited by small sample sizes, short duration of follow-up, having efficacy rather than safety as their primary outcome and that different methodologies were required to identify rarer adverse events. In contrast, randomised trials^13–17^ and cohort studies^18–20^ suggest that colchicine is protective against cardiovascular events, although a randomised trial of the cardiovascular benefits of colchicine specifically in people with gout is yet to be performed. There have been few studies of the effectiveness or side effects of NSAID prophylaxis against gout flares when initiating ULT, and existing recommendations are based on what is known about the risk of NSAID side effects in the wider literature.

We undertook two complementary matched retrospective cohort studies to determine the risk of adverse events severe enough to warrant seeking healthcare associated with (1) colchicine or (2) NSAID prophylaxis when initiating allopurinol in patients with gout in UK primary care.

## Methods

### Data source

We used data from the Clinical Practice Research Datalink (CPRD, December 2019) GOLD and Aurum datasets which have more than 3 and 13 million patients, respectively, currently registered and research acceptable. CPRD contains electronic, coded information collected during the course of routine primary healthcare, is representative of the UK population in terms of age, sex and ethnicity, and has been used extensively for primary care research.^21–23^ We used primary care data linked to the Hospital Episode Statistics (HES) admitted patient care dataset, which has coverage from April 1997 to the present.

### Study design

We performed two matched retrospective cohort studies. Cohort 1 compared the risk of adverse events in people with gout who received a prescription for colchicine when initiating allopurinol with those who did not receive prophylaxis. In cohort 2, people with gout who received a prescription for an NSAID when initiating allopurinol were compared with those who did not receive prophylaxis.

### Study population

We identified all permanently registered patients in GOLD and Aurum databases aged 18 years and over with a Read code for gout who received a new prescription for allopurinol between 1 April 1997 and 30 November 2016 (to allow HES linkage). The positive predictive value of a diagnosis of gout in CPRD has been shown to be 90%.^24^ The definition of ULT was restricted to allopurinol since this is by far the most commonly prescribed ULT in UK primary care, accounting for 99% of first ULT prescriptions.^25 26^

Practices can ‘migrate’ between clinical computing systems, and therefore, between the GOLD and Aurum databases. Records are ported from one system to the other and are therefore replicated. To account for this, individuals whose practices ‘migrated’ were removed from the GOLD dataset.

#### Exposure to colchicine or NSAID

For cohort 1, the exposed group was defined as those who received concomitant incident prescriptions for allopurinol and colchicine (on the same day, documented in the therapy record) and did not receive a prescription for NSAID or glucocorticoids in the preceding month. The index date (ie, start of time at risk) in the exposed group was the date of the concomitant incident prescriptions for allopurinol and colchicine. Each individual was followed up for 6 months or until the end of colchicine treatment, whichever was sooner. The end of treatment was defined as 56 days after the last colchicine prescription.

For cohort 2, the exposed group was defined similarly as having received concomitant incident prescriptions for allopurinol and an NSAID on the same day and did not receive a prescription for colchicine or glucocorticoids in the preceding month.

The potential unexposed group for both cohorts was defined as those who received a new prescription for allopurinol between 1 April 1997 and 30 November 2016 but did not concomitantly receive an incident prescription for colchicine, an NSAID or an oral glucocorticoid on the same day or in the preceding month.

#### Matching

Each exposed individual was matched 1:1 to an unexposed individual for age (within 3 years), gender, index date (within 3 years) and propensity score for receiving (1) colchicine or (2) NSAID. A propensity score is the probability (between 0 and 1) that an individual received (1) colchicine or (2) NSAID, given observed baseline characteristics. The observed characteristics in both cohorts were chronic kidney disease (CKD), Charlson Comorbidity Score,^27^ number of prescribed medications (simple count), number of gout consultations and hospital admission for gout, recorded during the year before the index date, plus prescriptions that may interact with colchicine recorded in the 30 days before the index date (statins, fibrates, verapamil, diltiazem, digoxin, amiodarone, oral ketoconazole and/or macrolide antibiotics) in cohort 1 and hypertension, dyslipidaemia, peptic ulcer disease (PUD), smoking (current) and prescription of anticoagulants in cohort 2. Matching was performed using a nearest neighbour approach with a calliper distance of 0.2 SD of the logit of the propensity score, in order to reduce the mean squared error of the exposure effect.^28^ Age, gender and index date were not included in the propensity score, as they were considered strong potential confounders and therefore matched separately.^29^ The covariate balance between exposed and unexposed individuals before and after matching was compared using standardised differences (defined as the difference in means or proportions divided by the respective SE).

### Outcomes

In cohort 1, incidence of the following adverse events was defined using Read/SNOMED codes in CPRD and International Classification of Diseases (ICD) codes in HES during the at-risk period of colchicine treatment: diarrhoea, nausea/vomiting, neuropathy, myalgia, myopathy, rhabdomyolysis, bone marrow suppression and myocardial infarction (MI). In cohort 2, we used the same approach to define MI, PUD, angina and acute kidney injury (AKI). In both cohorts, adverse events were analysed and reported separately, and all outcomes were also combined into a composite outcome, ‘any adverse event’.

Patients were excluded from the analysis of a particular outcome if they had a Read/ICD code for the outcome in question in the 3 months preceding the index date. It was assumed that the absence of a recorded adverse event meant that the patient did not consult for that condition or that if they did, the clinician did not think it of sufficient relevance to record in the coded data.

### Sample size calculation

An a priori feasibility count in CPRD GOLD identified 19 118 eligible individuals during the study period, 58% in practices with HES linkage. Hence, we conservatively estimated 19 000 eligible people in GOLD/AURUM would be available for analysis, providing 93% power to detect an HR 1.25 (1:1 matching, 5% significance level, annual incidence myalgia in the unexposed 2.5%).

### Statistical analysis

We quantified the absolute risk of adverse events in the exposed and unexposed groups in terms of events per 10 000 person-years with accompanying 95% CI. The association (HR, 95% CI) between exposure status (colchicine or NSAID prophylaxis) and the first occurrence of each outcome in CPRD or HES was investigated using a mixed-effect censoring-weighted Cox proportional hazards model adjusted for any matching factor with a standardised difference after matching >0.1. The assumption of proportional hazards was tested using Schoenfeld residuals. If necessary, time-varying covariates (for potential confounding factors) or an interaction of exposure status with time (in the case of the exposure of interest) was estimated. GOLD and Aurum datasets were analysed separately and then combined using two-stage individual patient data (IPD) meta-analysis using a fixed effects model as there was no clinical or methodological heterogeneity between databases, with an inverse variance approach to pool estimates.

#### Sensitivity analyses

To account for colchicine/NSAID prescriptions to treat flares in the unexposed group during follow-up, sensitivity analyses (1) excluded those in the unexposed group who received a prescription for colchicine/NSAID after the index date; (2) modelled colchicine/NSAID as a time-varying exposure. We also undertook a post hoc analysis excluding people who had ever had an MI to explore whether associations between colchicine prophylaxis and MI were influenced by having a prior history of MI.

Statistical analyses were conducted using Stata V.16.1 (StataCorp) and R V.4.0.5 (R Foundation for Statistical Computing, Vienna, Austria).

### Patient and public involvement

People living with gout informed our study design and recommended the inclusion of the composite outcome, risk of ‘any adverse event’. They helped us to interpret the study findings from a patient perspective, advised us on how to translate these into easily understandable messages for dissemination, and advised on our dissemination strategy.

## Results

### Cohort 1: colchicine exposed versus unexposed

A total of 13 945 individuals (2439 in CPRD GOLD, 11 506 in CPRD Aurum) with gout who initiated allopurinol with colchicine prophylaxis were matched to 13 945 who initiated without prophylaxis (mean age 64.0 (SD 14.6) years in GOLD and 63.7 (SD 14.9) years in Aurum; 78% male) (table 1). In both GOLD and Aurum, exposed and unexposed individuals were well matched for age and gender and did not appear to differ according to the number of prescribed medications, prescribed medications with the potential to interact with colchicine or NSAIDs, or the number of primary care consultations or hospital admissions for gout, although in CPRD GOLD the prevalences of CKD and Charlson Comorbidity Score were slightly higher in colchicine-exposed individuals than unexposed. Mean starting allopurinol dose (SD) was 178.9 (97.8) mg in GOLD and 171.9 (96.0) mg in Aurum in the colchicine-exposed individuals compared with 183.2 (98.6) mg in GOLD and 174.8 (96.8) in Aurum in unexposed individuals. Median duration of colchicine prophylaxis was 56 (IQR 42–78) days for GOLD and 86 (IQR 75–112) days for Aurum datasets.

**Table 1:** Baseline characteristics of matched colchicine exposed and unexposed groups in CPRD GOLD and Aurum in cohort 1

	CPRD GOLD			CPRD Aurum		
	Colchicine (n=2439)	No prophylaxis (n=2439)	Standardised difference†	Colchicine (n=11 506)	No prophylaxis (n=11 506)	Standardised difference†
Female gender	545 (22.3)	545 (22.3)	0.000	2495 (21.7)	2495 (21.7)	0.000
Age (year) (mean, SD)	64.0 (14.6)	64.0 (14.6)	0.001	63.7 (14.9)	63.8 (14.8)	0.001
Ever diagnosed with CKD stages 3–5	735 (30.1)	682 (28.0)	0.078	3052 (26.5)	2742 (23.8)	0.062
Previous vascular disease‡	848 (34.8)	790 (32.4)	0.050	3850 (33.5)	3617 (31.4)	0.043
Dyslipidaemia	942 (38.6)	964 (39.5)	0.019	4567 (39.7)	4513 (39.2)	0.010
Current smoker	268 (11.0)	242 (9.9)	0.065	1801 (15.7)	1631 (14.2)	0.005
Prescribed aspirin	604 (24.8)	623 (25.5)	0.018	2659 (23.1)	2743 (23.8)	0.017
Charlson score						
0	750 (30.8)	829 (34.0)	0.103	3675 (31.9)	3985 (34.6)	0.067
1	445 (18.2)	471 (19.3)		1958 (17.0)	1975 (17.2)	
2	401 (16.4)	391 (16.0)		1923 (16.7)	1916 (16.7)	
3	293 (12.0)	263 (10.8)		1392 (12.1)	1285 (11.2)	
≥4	550 (26.6)	485 (19.9)		2558 (22.2)	2345 (20.4)	
No of medications currently prescribed (quartile range)§						
Q1	531 (21.8)	634 (26.0)	0.104	2104 (18.3)	2507 (21.8)	0.091
Q2	540 (22.1)	535 (21.9)		3182 (27.7)	3037 (26.4)	
Q3	660 (27.1)	630 (25.8)		3124 (27.1)	2902 (25.2)	
Q4	708 (29.0)	640 (26.2)		3096 (26.9)	3060 (26.6)	
Potentially interacting medications						
Statin	542 (22.2)	532 (22.6)	0.009	2401 (20.9)	2406 (20.9)	0.001
Fibrate	16 (0.7)	7 (0.2)	0.053	62 (0.5)	74 (0.6)	0.014
Verapamil	16 (0.7)	11 (0.5)	0.027	49 (0.4)	42 (0.4)	0.010
Diltiazem	34 (1.4)	26 (1.1)	0.030	205 (1.8)	181 (1.6)	0.016
Digoxin	145 (5.9)	78 (3.9)	0.095	552 (4.8)	441 (3.8)	0.048
Amiodarone	21 (0.9)	11 (0.5)	0.051	72 (0.6)	61 (0.5)	0.013
Oral ketoconazole	* (0.0)	0 (0.0)	0.029	20 (0.2)	23 (0.2)	0.006
Macrolide antibiotic	29 (1.2)	22 (0.9)	0.028	107 (0.9)	117 (1.0)	0.009
No of primary care gout consultations in the last year						
0	1137 (46.6)	1163 (47.7)	0.047	5497 (47.8)	5066 (44.0)	0.090
1	789 (32.3)	762 (31.2)		3273 (28.4)	3336 (29.0)	
2	313 (12.8)	330 (13.5)		1354 (11.8)	1631 (14.2)	
3	104 (4.3)	104 (4.3)		669 (5.8)	718 (6.2)	
≥4	96 (3.9)	80 (3.3)		713 (6.2)	755 (6.6)	
Hospital admission for gout in the last year	71 (2.9)	43 (1.8)	0.076	352 (3.1)	232 (2.0)	0.066

n (%) unless otherwise stated.

*N<5.

†Standardised difference=difference in means or proportions divided by the respective SE.

‡Myocardial infarction, cardiac failure, peripheral vascular disease, cerebrovascular accident, transient ischaemic attack.

§Quartile ranges for number of medications currently prescribed: GOLD Q1 0–4, Q2 5–8, Q3 9–14, Q4 15–76. Aurum Q1 0–3, Q2 4–8, Q3 9–14, Q4 15–101.

CKD, chronic kidney disease; CPRD, Clinical Practice Research Datalink.

Following two-stage IPD meta-analysis combining GOLD and Aurum data, diarrhoea was the most common adverse event in the colchicine group, followed by nausea/vomiting, MI, neuropathy, myalgia and bone marrow suppression (table 2). Diarrhoea, MI, neuropathy, myalgia and bone marrow suppression were significantly more common with colchicine prophylaxis compared with no prophylaxis, but there was no statistically significant association between colchicine prophylaxis and nausea/vomiting. The incidence of having any adverse event per 10 000 person-years was 1292.3 (95% CI 1174.0 to 1422.4) in the colchicine-exposed group compared with 613.3 (95% CI 555.0 to 677.8) in the unexposed (HR 1.91; 95% CI 1.65 to 2.20). The total number of events (in exposed and unexposed groups in GOLD and Aurum) for myopathy and rhabdomyolysis were both <5, hence incidence and risk estimates were not calculated.

**Table 2:** Incidence rates per 10 000 person-years (95% CI) and risk of adverse events with colchicine exposure in CPRD GOLD and Aurum databases separately and combined using two-stage individual patient data meta-analysis

Adverse event	Colchicine			No prophylaxis			HR (95% CI)
	Event	Person-years	Incidence rate per 10000-person years (95% CI)	Event	Person-years	Incidence rate per 10 000 person-years (95% CI)	
Diarrhoea							
GOLD	75	0.0470	1604.9 (1280.5 to 2038.9)	69	0.1184	581.4 (459.3 to 746.9)	2.50 (1.72 to 3.61)
Aurum	191	0.3197	596.1 (517.6 to 690.3)	151	0.5600	270.4 (230.8 to 318.9)	2.12 (1.69 to 2.66)
Combined			784.4 (694.0 to 886.5)			341.9 (298.9 to 391.2)	2.22 (1.83 to 2.69)
Nausea and vomiting							
GOLD	9	0.0464	195.5 (103.9 to 414.8)	9	0.1169	77.3 (41.1 to 164.0)	2.50 (0.90 to 6.99)
Aurum	67	0.3189	209.7 (165.7 to 269.4)	80	0.5572	143.7 (115.8 to 180.6)	1.25 (0.89 to 1.76)
Combined			208.1 (165.4 to 261.7)			135.7 (109.8 to 167.6)	1.34 (0.97 to 1.85)
Bone marrow suppression							
GOLD	*	0.0463	64.9 (20.3 to 318.9)	*	0.1173	8.5 (0.2 to 47.5)	2.75 (0.28 to 26.55)
Aurum	16	0.3176	50.4 (31.4 to 86.4)	8	0.5567	14.4 (7.4 to 2.3)	3.38 (1.38 to 8.30)
Combined			51.9 (32.3 to 83.5)			13.9 (6.8 to 28.3)	3.29 (1.43 to 7.58)
Neuropathy							
GOLD	7	0.0464	151.4 (73.7 to 364.1)	*	0.1172	17.2 (3.7 to 172.2)	9.36 (1.85 to 47.45)
Aurum	*	0.3179	3.2 (0.1 to 17.5)	*	0.5565	3.6 (0.8 to 36.1)	0.86 (0.07 to 11.32)
Combined			110.8 (51.5 to 238.3)			7.9 (2.0 to 30.6)	4.75 (1.20 to 18.76)
Myalgia							
GOLD	13	0.0464	281.7 (166.8 to 515.8)	10	0.1175	85.7 (47.1 to 173.6)	4.80 (1.96 to 11.78)
Aurum	13	0.3182	40.9 (24.2 to 75.0)	13	0.5568	23.4 (13.8 to 42.9)	1.64 (0.74 to 3.66)
Combined			107.6 (72.1 to 160.4)			40.8 (26.6 to 62.6)	2.64 (1.45 to 4.81)
Myocardial infarction							
GOLD	12	0.0464	260.8 (151.0 to 492.0)	9	0.1171	77.4 (41.1 to 164.4)	2.15 (0.87 to 5.33)
Aurum	60	0.3179	189.0 (147.4 to 246.6)	69	0.5554	124.1 (98.4 to 158.9)	1.47 (1.02 to 2.11)
Combined			199.0 (157.2 to 251.9)			118.1 (94.1 to 148.0)	1.55 (1.10 to 2.17)
Any adverse event							
GOLD	123	0.0474	2584.2 (2158.7 to 3119.4)	106	0.1189	889.0 (732.7 to 1089.3)	2.62 (1.96 to 3.50)
Aurum	320	0.3209	997.6 (892.9 to 1118.2)	302	0.5594	540.4 (482.1 to 607.8)	1.72 (1.45 to 2.03)
Combined			1292.3 (1174.00 to 1422.4)			613.3 (555.0 to 677.8)	1.91 (1.65 to 2.20)

*N<5.

CPRD, Clinical Practice Research Datalink.

The sensitivity analysis which excluded unexposed individuals with a colchicine prescription after the index date produced similar findings (online supplemental table 1), although the association between colchicine prophylaxis and neuropathy was no longer statistically significant (HR 5.14; 95% CI 0.99 to 26.67). Similar associations were seen in the sensitivity analysis modelling colchicine exposure as time-varying, although those between colchicine prophylaxis and neuropathy, myalgia and bone marrow suppression were no longer statistically significant (online supplemental table 2). In the post hoc sensitivity analysis of the association between colchicine prophylaxis and MI which excluded people who had ever had an MI, findings were similar (HR 1.72; 95% CI 1.07 to 2.77) (online supplemental table 3).

10.1136/ard-2023-224154.supp1 Supplementary data

### Cohort 2: NSAID exposed versus unexposed

A total of 25 980 individuals (5293 in CPRD GOLD, 20 687 in CPRD Aurum) with gout who initiated allopurinol with NSAID prophylaxis were matched to 25 980 who initiated without prophylaxis (mean age 58.5 (SD 14.0) years in GOLD and 58.0 (SD 14.0) years in Aurum; 77% male) (table 3). In both GOLD and Aurum, exposed and unexposed individuals were well matched for age and gender and did not appear to differ according to CKD, PUD, hypertension, anticoagulant use, smoking, Charlson Comorbidity Score, number of prescribed medications or number of primary care consultations or hospital admissions for gout, although in both GOLD and Aurum the prevalence of dyslipidaemia was slightly lower in NSAID-exposed individuals than unexposed. Mean starting allopurinol dose (SD) was 204.7 (99.9) mg in GOLD and 197.5 (99.4) mg in Aurum in the NSAID-exposed individuals compared with 194.6 (99.9) mg in GOLD and 189.3 (99.4) in Aurum in unexposed individuals. Median duration of NSAID prophylaxis was 56 (IQR 56–84) days for GOLD and 84 (IQR 84–134) days for Aurum datasets.

**Table 3:** Baseline characteristics of matched NSAID exposed and unexposed groups in CPRD GOLD and Aurum in cohort 2

	CPRD GOLD			CPRD Aurum		
	NSAID (n=5293)	No prophylaxis (n=5293)	Standardised difference*	NSAID (n=20 687)	No prophylaxis (n=20 687)	Standardised difference*
Female gender	724 (13.7)	724 (13.7)	0.000	2674 (12.9)	2674 (12.9)	0.000
Age (mean, SD)	58.5 (14.0)	58.60 (13.9)	0.009	58.0 (13.9)	58.1 (13.8)	0.004
Ever diagnosed with CKD stages 3–5	463 (8.7)	568 (10.7)	0.052	1497 (7.2)	1811 (8.8)	0.056
Ever diagnosed with PUD	6 (0.1)	6 (0.1)	0.020	58 (0.3)	84 (0.4)	0.022
Hypertension	2292 (43.3)	2611 (49.3)	0.136	8557 (41.4)	9722 (47.0)	0.114
Previous vascular disease†	965 (18.2)	1113 (21.0)	0.070	4344 (21.0)	3545 (17.1)	0.098
Dyslipidaemia	1128 (21.3)	1301 (24.6)	0.078	4472 (21.6)	5056 (24.4)	0.067
Current smoker	779 (14.7)	717 (13.5)	0.034	3614 (17.5)	3637 (17.6)	0.003
Prescribed aspirin	853 (16.1)	906 (17.1)	0.027	2998 (14.5)	3317 (16.0)	0.043
Prescribed anticoagulant	41 (0.8)	95 (1.8)	0.093	256 (1.2)	429 (2.1)	0.066
Charlson score						
0	2823 (53.3)	2642 (49.1)	0.104	11 402 (55.1)	10 437 (50.5)	0.103
1	1073 (20.3)	1129 (21.3)		4093 (19.8)	4376 (21.2)	
2	687 (13.0)	677 (12.8)		2566 (12.4)	2712 (13.1)	
3	338 (6.4)	370 (7.0)		1214 (5.9)	1390 (6.7)	
≥4	372 (7.0)	475 (9.0)		1412 (6.8)	1772 (8.6)	
No of medications currently prescribed (quartile range)‡						
Q1	2388 (45.1)	2068 (39.1)	0.141	8000 (38.7)	7034 (34.0)	0.124
Q2	1320 (24.9)	1446 (27.3)		6887 (33.3)	6842 (33.1)	
Q3	1004 (19.0)	1068 (20.2)		3295 (15.9)	3637 (17.6)	
Q4	581 (11.0)	711 (13.4)		2505 (12.1)	3174 (15.3)	
No of primary care gout consultations in the last year						
0	3175 (60.0)	2719 (51.4)	0.166	12 405 (60.0)	10 630 (51.4)	0.173
1	1428 (27.0)	1656 (31.3)		4923 (23.8)	5989 (29.0)	
2	464 (8.8)	626 (11.8)		1765 (8.5)	2140 (10.3)	
3	144 (2.7)	185 (3.5)		756 (3.7)	927 (4.5)	
≥4	82 (1.5)	107 (2.0)		838 (4.1)	1001 (4.8)	
Hospital admission for gout in the last year	60 (1.1)	78 (1.5)	0.012	251 (1.2)	285 (1.4)	0.015

n (%) unless otherwise stated.

*Standardised difference=difference in means or proportions divided by the respective SE.

†Myocardial infarction, cardiac failure, peripheral vascular disease, cerebrovascular accident or transient ischaemic attack.

‡Quartile ranges for number of medications currently prescribed: GOLD Q1 0–4, Q2 5–8, Q3 9–14, Q4 15–72. Aurum Q1 0–3, Q2 4–8, Q3 9–13, Q4 14–69.

CKD, chronic kidney disease; CPRD, Clinical Practice Research Datalink; NSAID, non-steroidal anti-inflammatory drug; PUD, peptic ulcer disease.

Following two-stage IPD meta-analysis combining GOLD and Aurum data, the most common adverse event in the NSAID group was angina (incidence rate per 10 000 person-years 466.6; 95% CI 417.2 to 521.8), followed by AKI (160.7; 95% CI 132.9 to 194.5), MI (156.7; 95% CI 129.7 to 189.4) and PUD (81.7; 95% CI 62.4 to 106.9) (table 4). Angina (HR 1.60; 95% CI 1.37 to 1.86), AKI (1.56; 95% CI 1.20 to 2.03), MI (1.89; 95% CI 1.44 to 2.48) and PUD (1.67; 95% CI 1.14 to 2.44) were significantly more common with NSAID prophylaxis compared with no prophylaxis. The incidence of having any adverse event per 10 000 person-years was 740.2 (95% CI 676.3 to 810.2) in the NSAID-exposed group compared with 532.7 (95% CI 492.0 to 576.8) in the unexposed (HR 1.63; 95% CI 1.44 to 1.85).

**Table 4:** Incidence rates per 10 000 person-years (95% CI) and risk of adverse events with NSAID exposure in CPRD GOLD and Aurum databases separately and combined using 2-stage individual patient data meta-analysis

Adverse event	NSAID			No prophylaxis			HR (95% CI)
	Event	Person-years	Incidence rate per 10000-person years (95% CI)	Event	Person-years	Incidence rate per 10000-person years (95% CI)	
AKI							
GOLD	26	0.1053	242.9 (166.1 to 370.0)	29	0.2576	110.9 (77.5 to 164.4)	2.11 (1.17 to 3.81)
Aurum	86	0.6021	142.5 (115.4 to 178.0)	122	1.0130	120.1 (100.5 to 144.7)	1.45 (1.08 to 1.94)
Combined			160.7 (132.9 to 194.5)			118.3 (100.4 to 139.4)	1.56 (1.20 to 2.03)
Angina							
GOLD	63	0.1046	604.4 (472.3 to 786.7)	93	0.2553	362.7 (295.6 to 450.1)	1.92 (1.35 to 2.74)
Aurum	261	0.5959	438.7 (388.2 to 497.8)	343	1.0008	342.3 (307.4 to 382.3)	1.53 (1.30 to 1.82)
Combined			466.6 (417.2 to 521.8)			346.5 (314.6 to 381.7)	1.60 (1.37 to 1.86)
Myocardial infarction							
GOLD	20	0.1056	190.7 (124.8 to 306.5)	31	0.2581	121.7 (86.5 to 177.0)	1.68 (0.91 to 3.10)
Aurum	90	0.6021	150.3 (122.6 to 186.2)	94	1.0130	92.3 (75.6 to 114.0)	1.95 (1.44 to 2.64)
Combined			156.7 (129.7 to 189.4)			98.9 (82.7 to 118.2)	1.89 (1.44 to 2.48)
Peptic ulcer disease							
GOLD	15	0.1054	143.1 (87.8 to 249.9)	8	0.2580	31.2 (15.9 to 70.0)	8.52 (3.38 to 21.50)
Aurum	40	0.6024	66.7 (49.4 to 92.5)	61	1.0130	60.6 (47.4 to 78.6)	1.20 (0.80 to 1.82)
Combined			81.7 (62.4 to 106.9)			56.5 (44.5 to 71.8)	1.67 (1.14 to 2.44)
Any adverse event							
GOLD	103	0.1043	984.3 (809.3 to 1209.6)	137	0.2546	536.6 (452.3 to 641.6)	2.18 (1.63 to 2.90)
Aurum	408	0.5930	688.7 (623.4 to 763.0)	529	0.9953	531.7 (486.7 to 581.9)	1.53 (1.33 to 1.75)
Combined			740.2 (676.3 to 810.2)			532.7 (492.0 to 576.8)	1.63 (1.44 to 1.85)

AKI, acute kidney injury; CPRD, Clinical Practice Research Datalink; NSAID, non-steroidal anti-inflammatory drug.

The sensitivity analysis which excluded unexposed individuals with an NSAID prescription after the index date produced similar findings, although the HRs attenuated slightly, and the association between NSAID prophylaxis and PUD was no longer statistically significant (HR 1.43; 95% CI 0.90 to 2.28) (online supplemental table 4). In the sensitivity analysis modelling NSAID exposure as time-varying, AKI (1.68; 95% CI 0.95 to 2.97) and PUD (1.57; 95% CI 0.66 to 3.74) were no longer statistically significant (online supplemental table 5).

## Discussion

This is the first large observational study to quantify the incidence of adverse events from colchicine and NSAID prophylaxis when initiating allopurinol for gout, using primary care data linked to hospital admission records to capture rare but potentially serious side effects outside the confines of a randomised controlled trial (RCT). Compared with initiation of allopurinol without prophylaxis, we found that diarrhoea, MI, neuropathy, myalgia and bone marrow suppression were more common with colchicine prophylaxis, and angina, AKI, MI and PUD were more common with NSAID prophylaxis. Other than diarrhoea for colchicine and angina for NSAID, the incidence of individual adverse events was low (<200 per 10 000 treated patient-years), although the number needed to harm in relation to any adverse event was 14.7 for colchicine, driven mainly by diarrhoea, and 48.1 for NSAID.

Previous studies have demonstrated the clinical and cost-effectiveness of colchicine prophylaxis when initiating ULT.^10 11^ There have been no randomised trials or large observational studies of NSAID prophylaxis, other than with azapropazone,^30^ which is rarely used in clinical practice today. Our findings accord with a small RCT of 43 participants that found 38% of participants experienced diarrhoea following prophylaxis with colchicine 0.6 mg two times per day.^10 31^ A more recent systematic review examined colchicine-related adverse events reported in 35 RCTs (4225 participants randomised to receive colchicine), although the indications for colchicine were broad including cirrhosis, gout, pericarditis, osteoarthritis and Behcet’s syndrome, and the 5 gout RCTs included three of a short course of colchicine to treat gout flares rather than longer courses for prophylaxis.^12^ Similar to our findings, diarrhoea was the most common adverse event, affecting 17.9% of participants in the colchicine arms, while muscle (4.2%) and haematological (0.6%) adverse events were uncommon. No included studies reported rhabdomyolysis or neurological adverse events. Gastrointestinal symptoms can also be caused by allopurinol. Although the allopurinol starting dose was similar between the colchicine/NSAID prophylaxis and no prophylaxis groups, the dose was higher (mean 170–204 mg per day) than is currently recommended in gout management guidelines.^4–7^ Our findings concerning the incidence of adverse events from NSAID prophylaxis are consistent with the existing literature concerning NSAID use for other indications.^32–34^

In light of several large RCTs which have shown cardiovascular benefits of colchicine in people with coronary heart disease or post-MI,^13–17^ the most surprising finding of our study is that MI was more common in people initiating allopurinol with colchicine prophylaxis than those initiating without prophylaxis. The dose of colchicine used in these trials was 0.5 mg daily, which is consistent with that recommended for prophylaxis in gout management guidelines and commonly used in clinical practice.^4 5^ However, the trial participants had a history of either coronary heart disease or recent MI and were, therefore, likely to be at higher risk of future cardiovascular events than our study population, only one-third of whom had a prior history of vascular disease. Furthermore, the average colchicine treatment duration in these trials ranged from 19.5 to 36 months, which is considerably longer than the median duration of colchicine prophylaxis of 2–3 months in our study. It is possible that the typical duration of colchicine prophylaxis in current primary care practice is insufficient to realise the cardiovascular benefits seen with longer therapy in these trials, although two previous cohort studies have shown cardiovascular benefits of colchicine in people with gout. In a cohort study undertaken in male US veterans with gout, colchicine use was associated with reduced incidence of coronary artery disease compared with colchicine non-users, although the median duration of prophylaxis was 23 months which is longer than in our primary care-based study.^20^ A hospital-based cohort study found fewer cardiovascular events in colchicine users with gout than non-users, although there was no clear gradient of effect according to duration of use.^19^ Despite matching on propensity to receive prophylactic colchicine or NSAID to reduce confounding by indication, residual confounding remains possible in our study as a result of not including all relevant prognostic factors or unknown factors biasing the results.^35^ However, the calculation of an E-value^36^ suggests that to attenuate the HR of 1.55 (95% CI 1.10 to 2.17) for the association between colchicine prophylaxis and MI, an unmeasured confounder (or combination of confounders) would need to confer an increased hazard of at least 147%. It seems unlikely that we would have missed a confounder of this magnitude.

The main strengths of our study are the large sample size and use of primary care consultation and prescription data linked to hospital records over a period of 20 years to derive a comprehensive, high-quality dataset from everyday clinical practice. Several limitations are worthy of acknowledgement. First, gout was ascertained according to a clinical diagnosis in primary care rather than classification criteria or synovial fluid microscopy following joint aspiration. However, a coded gout diagnosis in CPRD has a positive predictive value of 90%^24^ and these patients were being managed by their general practitioner as though they did have gout. Second, use of CPRD/HES data permitted us to consider only adverse events severe enough to warrant consultation or resulting in hospitalisation and hence milder adverse events such as gastrointestinal symptoms may have been missed. Despite the size of our sample and linkage to hospital admissions data, coded occurrences of myopathy and rhabdomyolysis remained rare. A further caveat is the observational design which risks misclassification of exposure status, although we undertook sensitivity analyses to explore possible effects of colchicine/NSAID prescriptions in the unexposed group, finding similar results to the main analysis. We also could not ascertain use of over-the-counter NSAIDs or compare the effects of individual NSAIDs or colchicine dosing. Finally, from these observational data, we cannot make causal inferences. However, we did carry out propensity score matching to, as far as possible, reduce the risk of confounding by indication.

We found that adverse events were more common in people who initiated allopurinol with flare prophylaxis than those initiating without, although serious adverse events were uncommon, providing reassurance for people with gout and for clinicians. Future research is needed to determine which patients are at greatest risk of adverse events from prophylaxis and whether the cardiovascular benefits of colchicine reported in RCTs of people at high risk of cardiovascular events because of a prior history of coronary heart disease also apply to people with gout. Our findings will provide much-needed information about the safety of flare prophylaxis that can inform treatment decisions and the choice between colchicine or NSAID for prophylaxis when initiating allopurinol, directly benefiting people with gout and their clinicians.

## Data Availability

Data may be obtained from a third party and are not publicly available. The data were obtained from the Clinical Practice Research Datalink (CPRD). CPRD data governance does not allow us to distribute patient data to other parties. Researchers may apply for data access at http://www.CPRD.com/. Code lists for gout, medications, confounding variables and adverse events are publicly available on the Keele Research Repository: https://doi.org/10.21252/9fpd-dd05.
