# Supplementary material for: Safety of colchicine and NSAID prophylaxis when initiating urate-lowering therapy for gout: propensity score-matched cohort studies in the UK Clinical Practice Research Datalink
Source: Ann Rheum Dis. 2023 Oct 3;82(12):1618–25. doi: 10.1136/ard-2023-224154 (PMC10646835; doi:10.1136/ard-2023-224154)
Supplement: Supplementary data [file ard-2023-224154supp001.pdf]

Supplementary Table 1 Sensitivity analysis excluding unexposed individuals with a colchicine prescription after the index date: incidence rates per 10000 person-years (95% CI) and risk of adverse events with colchicine exposure in CPRD GOLD and Aurum databases separately and combined using 2-stage individual patient data meta-analysis

Adverse event	Colchicine			No prophylaxis			Hazard ratio (95%CI)
	Event	Person-years	Incidence rate per 10000-person years (95%CI)	Event	Person-years	Incidence rate per 10000 person-years (95%CI)	
**Diarrhoea**							
GOLD	65	0.0404	1608.76 (1261.42, 2084.16)	56	0.1020	552.12 (425.62, 729.41)	2.34 (1.57, 3.49)
Aurum	161	0.2681	600.27 (514.76, 704.40)	136	0.4688	290.13 (245.53, 345.39)	1.90 (1.49, 2.42)
Combined			*791.61 (693.02, 904.22)*			*348.84 (302.01, 402.92)*	*2.01 (1.63, 2.47)*
**Nausea and vomiting**							
GOLD	8	0.0398	202.48 (103.41, 454.15)	8	0.1007	79.84 (40.78, 179.05)	2.41 (0.80, 7.23)
Aurum	56	0.2674	208.47 (161.14, 274.69)	69	0.4665	147.70 (117.10, 189.07)	1.12 (0.73, 1.72)
Combined			*207.77 (161.67, 267.02)*			*139.32 (110.93, 174.98)*	*1.24 (0.83, 1.85)*
**Bone marrow suppression**							
GOLD	*	0.0399	50.20 (10.82, 504.00)	*	0.1008	9.95 (0.25, 55.27)	1.85 (0.17, 20.55)
Aurum	10	0.2665	37.57 (20.64, 76.15)	7	0.4661	15.04 (7.32, 36.20)	2.80 (1.00, 7.86)
Combined			*38.72 (20.87, 71.83)*			*14.55 (6.76, 31.30)*	*2.63 (1.02, 6.78)*
**Neuropathy**							
GOLD	7	0.0398	176.54 (85.96, 424.37)	*	0.1008	10.00 (0.25, 55.27)	17.39 (2.04, 147.93)
Aurum	*	0.2664	3.76 (0.10, 20.91)	*	0.4659	4.29 (0.93, 43.14)	0.88 (0.07, 11.55)
Combined			*128.76 (59.92, 276.69)*			*5.70 (1.19, 27.22)*	*5.14 (0.99, 26.67)*
**Myalgia**							
GOLD	11	0.0400	276.63 (156.44, 538.55)	9	0.1009	89.75 (47.68, 190.37)	4.71 (1.81, 12.26)
Aurum	12	0.2666	45.07 (26.20, 85.03)	11	0.4662	23.64 (13.35, 46.10)	1.77 (0.75, 4.16)
Combined			*107.13 (69.90, 164.20)*			*42.79 (26.97, 67.90)*	*2.73 (1.44, 5.18)*
**Myocardial infarction**							
GOLD	10	0.0398	253.14 (138.98, 512.94)	8	0.1008	79.92 (40.79, 189.38)	2.00 (0.75, 5.35)
Aurum	52	0.2666	194.77 (149.11, 259.47)	55	0.4649	117.71 (90.76, 155.53)	1.46 (0.98, 2.18)
Combined			*202.71 (157.09, 261.59)*			*112.51 (87.35, 144.91)*	*1.53 (1.06, 2.21)*

**Any adverse event**							
GOLD	109	0.0409	2664.73 (2201.61, 3255.57)	90	0.1025	875.11 (709.51, 1091.88)	2.56 (1.87, 3.49)
Aurum	268	0.2694	993.02 (879.74, 1125.19)	259	0.4684	553.14 (489.05, 628.12)	1.59 (1.33, 1.91)
Combined			1313.73 (1183.8, 1457.93)			620.95 (557.26, 691.92)	1.79 (1.53, 2.09)

CI, confidence interval; CPRD, Clinical Practice Research Datalink
*N<5

Supplementary Table 2 Sensitivity analysis modelling colchicine as a time-varying exposure: incidence rates per 10000 person-years (95% CI) and risk of adverse events with colchicine exposure in CPRD GOLD and Aurum databases separately and combined using 2-stage individual patient data meta-analysis

Adverse event	Colchicine			No prophylaxis			Hazard ratio (95%CI)
	Event	Person-years	Incidence rate per 10000-person years (95%CI)	Event	Person-years	Incidence rate per 10000 person-years (95%CI)	
**Diarrhoea**							
GOLD	75	0.0470	1604.93 (1280.45, 2038.92)	69	0.1184	581.36 (459.32, 746.86)	2.65 (1.24, 5.69)
Aurum	191	0.3197	596.13 (517.55, 690.30)	151	0.5600	270.35 (230.77, 318.86)	2.42 (1.52, 3.86)
Combined			*784.38 (693.98, 886.54)*			*341.91 (298.85, 391.17)*	*2.48 (1.67, 3.69)*
**Nausea and vomiting**							
GOLD	9	0.0464	195.51 (103.87, 414.75)	9	0.1169	77.33 (41.09, 164.03)	0.25 (0.00, 29.98)
Aurum	67	0.3189	209.65 (165.66, 269.37)	80	0.5572	143.72 (115.84, 180.58)	1.29 (0.58, 2.91)
Combined			*208.05 (165.41, 261.68)*			*135.65 (109.80, 167.58)*	*1.23 (0.56, 2.74)*
**Bone marrow suppression**							
GOLD	*	0.0463	64.89 (20.34, 318.92)	*	0.1173	8.54 (0.22, 47.50)	8132.00 (0.01, 8.08E+09)
Aurum	16	0.3176	50.40 (31.39, 86.35)	8	0.5567	14.40 (7.35, 32.31)	2.58 (0.33, 20.23)
Combined			*51.94 (32.30, 83.50)*			*13.88 (6.80, 28.34)*	*3.07 (0.40, 23.57)*
**Neuropathy**							
GOLD	7	0.0464	151.44 (73.73, 364.11)	*	0.1172	17.15 (3.70, 172.23)	5.57 (0.07, 414.56)
Aurum	*	0.3179	3.15 (0.08, 17.53)	*	0.5565	3.59 (0.77, 36.12)	5.31 (0.25, 114.65)
Combined			*110.83 (51.54, 238.33)*			*7.85 (2.02, 30.55)*	*5.40 (0.44, 65.85)*
**Myalgia**							
GOLD	13	0.0464	281.74 (166.80, 515.81)	10	0.1175	85.66 (47.05, 173.55)	4.49 (0.23, 88.17)
Aurum	13	0.3182	40.92 (24.21, 75.01)	13	0.5568	23.39 (13.84, 42.89)	2.18 (0.48, 9.87)
Combined			*107.56 (72.14, 160.39)*			*40.82 (26.62, 62.59)*	*2.53 (0.66, 9.72)*
**Myocardial infarction**							
GOLD	12	0.0464	260.84 (150.96, 492.01)	9	0.1171	77.44 (41.12, 164.37)	4.99 (0.49, 50.49)

Aurum	60	0.3179	189.03 (147.42, 246.57)	69	0.5554	124.14 (98.43, 158.90)	3.39 (1.53, 7,45)
Combined			*198.98 (157.18, 251.90)*			*118.05 (94.14, 148.03)*	*3.52 (1.66, 7.44)*
**Any adverse event**							
GOLD	123	0.0474	2584.22 (2158.70, 3119.36)	106	0.1189	889.00 (732.66, 1089.27)	3.38 (1.76, 6.48)
Aurum	320	0.3209	997.62 (892.92, 1118.16)	302	0.5594	540.37 (482.10, 607.75)	2.21 (1.54, 3.16)
Combined			*1292.25 (1174.00, 1422.42)*			*613.31 (554.95, 677.81)*	*2.44 (1.78, 3.34)*

CI, confidence interval; CPRD, Clinical Practice Research Datalink
*N<5

Supplementary Table 3 Sensitivity analysis excluding individuals who had had a myocardial infarction ever: incidence rates per 10000 person-years (95% CI) and risk of myocardial infarction with colchicine exposure in CPRD GOLD and Aurum databases separately and combined using 2-stage individual patient data meta-analysis

**Adverse event**	**Colchicine**			**No prophylaxis**			**Hazard ratio (95%CI)**
	Event	Person-years	Incidence rate per 10000-person years (95%CI)	Event	Person-years	Incidence rate per 10000 person-years (95%CI)	
**Myocardial infarction**							
GOLD	7	0.0462	152.38 (74.13, 366.68)	5	0.1168	43.02 (18.16, 128.40)	2.69 (0.79, 9.17)
Aurum	32	0.3164	99.92 (71.03, 145.26)	33	0.5532	60.41 (43.38, 86.75)	1.59 (0.95, 2.66)
Combined			*107.21 (77.35, 148.61)*			*58.17 (41.96, 80.63)*	*1.72 (1.07, 2.77)*

CI, confidence interval; CPRD, Clinical Practice Research Datalink

Supplementary Table 4 Sensitivity analysis excluding unexposed individuals with a NSAID prescription after the index date: incidence rates per 10000 person-years (95% CI) and risk of adverse events with NSAID exposure in CPRD GOLD and Aurum databases separately and combined using 2-stage individual patient data meta-analysis

**Adverse event**	**NSAID**			**No prophylaxis**			**Hazard ratio (95%CI)**
	Event	Person-years	Incidence rate per 10000-person years (95%CI)	Event	Person-years	Incidence rate per 10000 person-years (95%CI)	
**AKI**							
GOLD	15	0.0683	224.3 (137.5, 392.1)	21	0.1659	123.8 (81.0, 199.1)	2.22 (1.06, 4.63)
Aurum	58	0.4126	140.7 (109.0, 185.0)	99	0.6915	143.1 (117.5, 176.1)	1.24 (0.88, 1.74)
Combined			*154.7 (122.2, 195.9)*			*139.6 (116.1, 167.9)*	*1.37 (1.01, 1.87)*
**Angina**							
GOLD	39	0.0680	574.0 (419.7, 806.6)	66	0.1644	404.1 (317.5, 522.6)	1.52 (0.99, 2.33)
Aurum	190	0.4088	464.3 (402.4, 538.8)	279	0.6832	408.0 (362.2, 461.4)	1.30 (1.07, 1.58)
Combined			*481.0 (421.0, 549.6)*			*407.3 (365.3, 454.1)*	*1.34 (1.12, 1.59)*
**Myocardial infarction**							
GOLD	12	0.0688	175.6 (101.6, 331.1)	26	0.1666	158.1 (108.9, 238.6)	1.01 (0.49, 2.12)
Aurum	69	0.4127	167.3 (132.7, 214.1)	79	0.6918	114.6 (92.2, 144.2)	1.76 (1.26, 2.46)
Combined			*168.5 (135.0, 210.3)*			*124.0 (102.1, 150.5)*	*1.60 (1.18, 2.17)*
**Peptic ulcer disease**							
GOLD	11	0.0685	161.4 (91.2, 314.5)	6	0.1664	36.3 (16.6, 95.7)	7.39 (2.55, 21.39)
Aurum	24	0.4127	58.5 (39.7, 89.9)	43	0.6914	62.6 (46.8, 85.7)	0.98 (0.59, 1.64)
Combined			*79.6 (56.6, 112.0)*			*59.1 (44.4, 78.6)*	*1.43 (0.90, 2.28)*
**Any adverse event**							
GOLD	65	0.0679	962.8 (753.6, 1250.2)	99	0.1639	605.8 (495.8, 748.2)	1.84 (1.30, 2.59)
Aurum	294	0.4065	722.8 (642.7, 815.8)	424	0.6786	624.4 (565.7, 691.0)	1.32 (1.13, 1.54)
Combined			*761.4 (683.6, 848.1)*			*620.8 (567.4, 679.3)*	*1.39 (1.21, 1.61)*

AKI, acute kidney injury; CI, confidence interval; CPRD, Clinical Practice Research Datalink; NSAID, non-steroidal anti-inflammatory drug

Supplementary Table 5 Sensitivity analysis modelling NSAID as a time-varying exposure: incidence rates per 10000 person-years (95% CI) and risk of adverse events with NSAID exposure in CPRD GOLD and Aurum databases separately and combined using 2-stage individual patient data meta-analysis

**Adverse event**	**NSAID**			**No prophylaxis**			**Hazard ratio (95%CI)**
	Event	Person-years	Incidence rate per 10000-person years (95%CI)	Event	Person-years	Incidence rate per 10000 person-years (95%CI)	
**AKI**							
GOLD	26	0.1053	242.9 (166.1, 370.0)	29	0.2576	110.9 (77.5, 164.4)	1.12 (0.26, 5.34)
Aurum	86	0.6021	142.5 (115.4, 178.0)	122	1.0130	120.1 (100.5, 144.7)	1.78 (0.96, 3.30)
Combined			*160.7 (132.9, 194.5)*			*118.3 (100.4, 139.4)*	*1.68 (0.95, 2.97)*
**Angina**							
GOLD	63	0.1046	604.4 (472.3, 786.7)	93	0.2553	362.7 (295.6, 450.1)	4.42 (2.13, 9.18)
Aurum	261	0.5959	438.7 (388.2, 497.8)	343	1.0008	342.3 (307.4, 382.3)	3.42 (2.36, 4.97)
Combined			*466.6 (417.2, 521.8)*			*346.5 (314.6, 381.7)*	*3.61 (2.59, 5.03)*
**Myocardial infarction**							
GOLD	20	0.1056	190.7 (124.8, 306.5)	31	0.2581	121.7 (86.5, 177.0)	3.30 (0.94, 11.59)
Aurum	90	0.6021	150.3 (122.6, 186.2)	94	1.0130	92.3 (75.6, 114.0)	5.17 (2.43, 11.02)
Combined			*156.7 (129.7, 189.4)*			*98.9 (82.7, 118.2)*	*4.59 (2.40, 8.77)*
**Peptic ulcer disease**							
GOLD	15	0.1054	143.1 (87.8, 249.9)	8	0.2580	31.2 (15.9, 70.0)	10.84 (0.71, 165.36)
Aurum	40	0.6024	66.7 (49.4, 92.5)	61	1.0130	60.6 (47.4, 78.6)	1.26 (0.51, 3.15)
Combined			*81.7 (62.4, 106.9)*			*56.5 (44.5, 71.8)*	*1.57 (0.66, 3.74)*
**Any adverse event**							
GOLD	103	0.1043	984.3 (809.3, 1209.6)	137	0.2546	536.6 (452.3, 641.6)	3.98 (2.20, 7.20)
Aurum	408	0.5930	688.7 (623.4, 763.0)	529	0.9953	531.7 (486.7, 581.9)	2.91 (2.13, 3.97)
Combined			*740.2 (676.3, 810.2)*			*532.7 (492.0, 576.8)*	*3.11 (2.36, 4.09)*

AKI, acute kidney injury; CI, confidence interval; CPRD, Clinical Practice Research Datalink; NSAID, non-steroidal anti-inflammatory drug
